# Supplementary material for: Impact of oral probiotic Lactobacillus acidophilus vaccine strains on the immune response and gut microbiome of mice
Source: PLoS One. 2019 Dec 12;14(12):e0225842. doi: 10.1371/journal.pone.0225842 (PMC6907787; doi:10.1371/journal.pone.0225842)
Supplement: S1 Fig — (PDF) [file pone.0225842.s001.pdf]

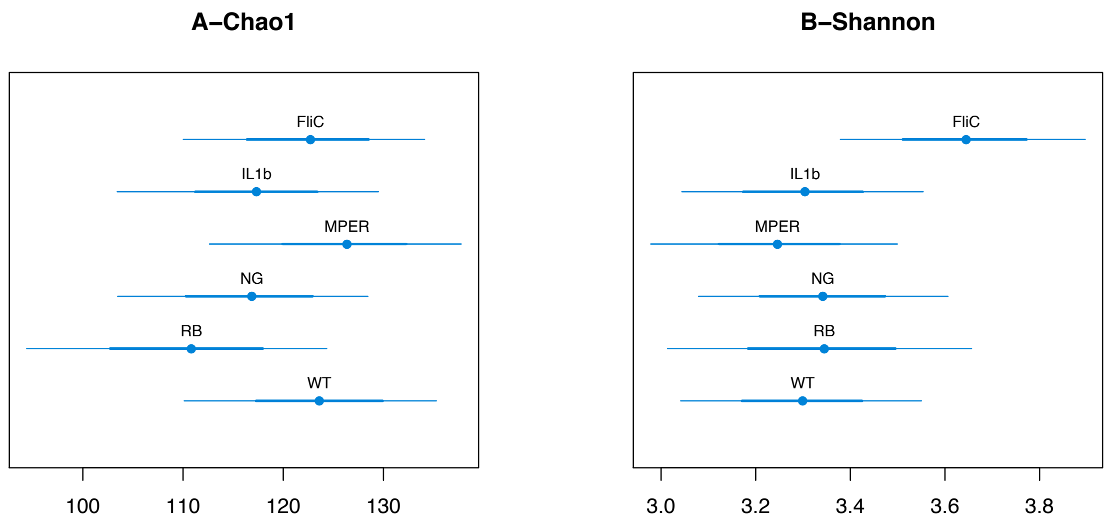

S1 Fig. (A) 95% credibility intervals of the expected Chao1 richness treatment levels for cecal samples. (B) 95% credibility intervals of the expected Shannon diversity index per treatment levels for cecal samples.
