# Supplementary material for: Impact of oral probiotic Lactobacillus acidophilus vaccine strains on the immune response and gut microbiome of mice
Source: PLoS One. 2019 Dec 12;14(12):e0225842. doi: 10.1371/journal.pone.0225842 (PMC6907787; doi:10.1371/journal.pone.0225842)
Supplement: S2 Fig — (PDF) [file pone.0225842.s002.pdf]

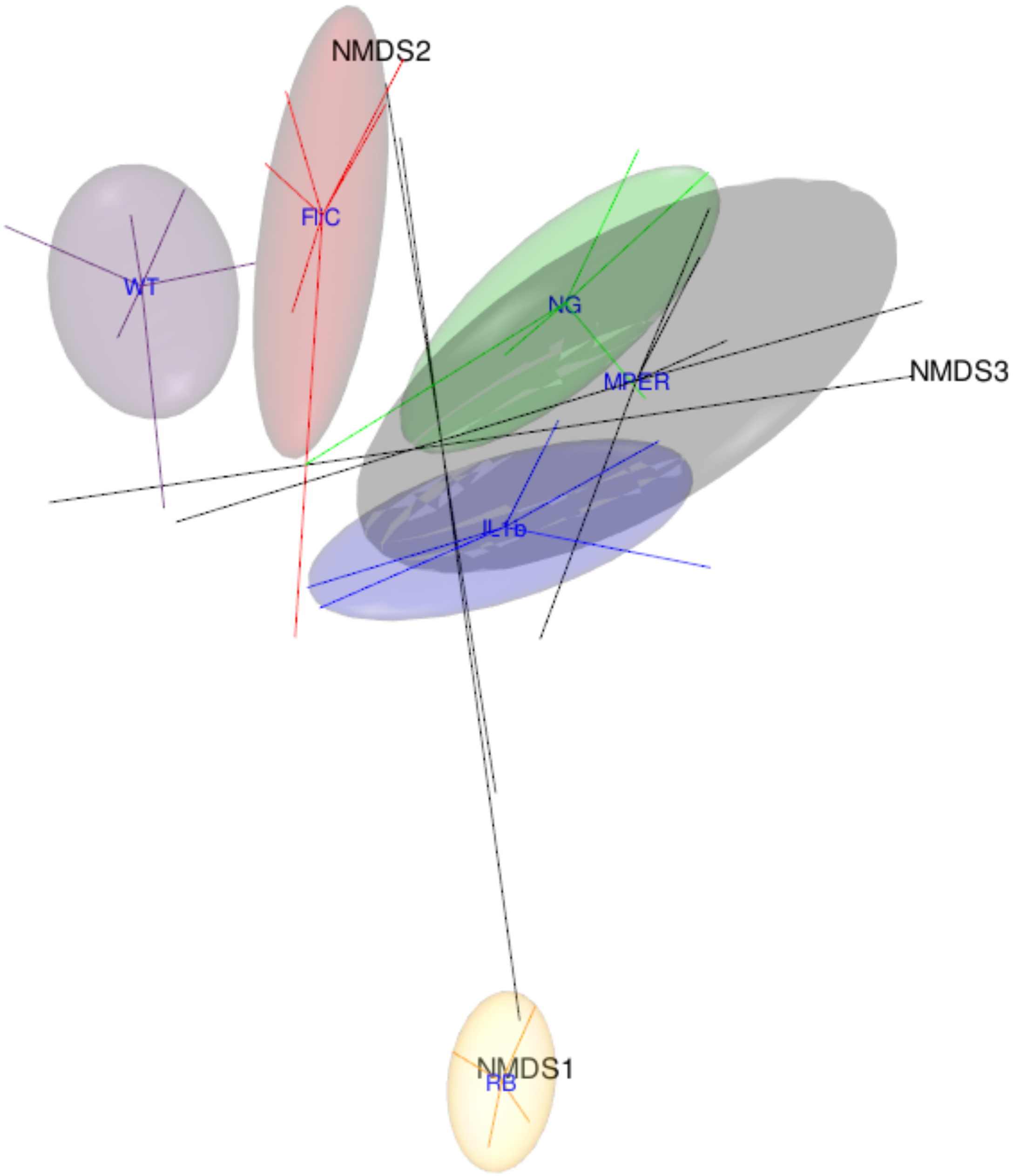

S2 Fig.: Three-dimensional (3D) nonmetric multidimensional scaling (NMDS) ordination plot of the beta diversity of cecal samples measured using the Bray-Curtis distance and aggregated by treatment level. The figure highlights the different data points as tips of the star shapes emitted from the centroids representing the treatment levels and the associated 95% confidence ellipsoids.
