# Supplementary material for: Impact of oral probiotic Lactobacillus acidophilus vaccine strains on the immune response and gut microbiome of mice
Source: PLoS One. 2019 Dec 12;14(12):e0225842. doi: 10.1371/journal.pone.0225842 (PMC6907787; doi:10.1371/journal.pone.0225842)
Supplement: S3 Fig — (PDF) [file pone.0225842.s003.pdf]

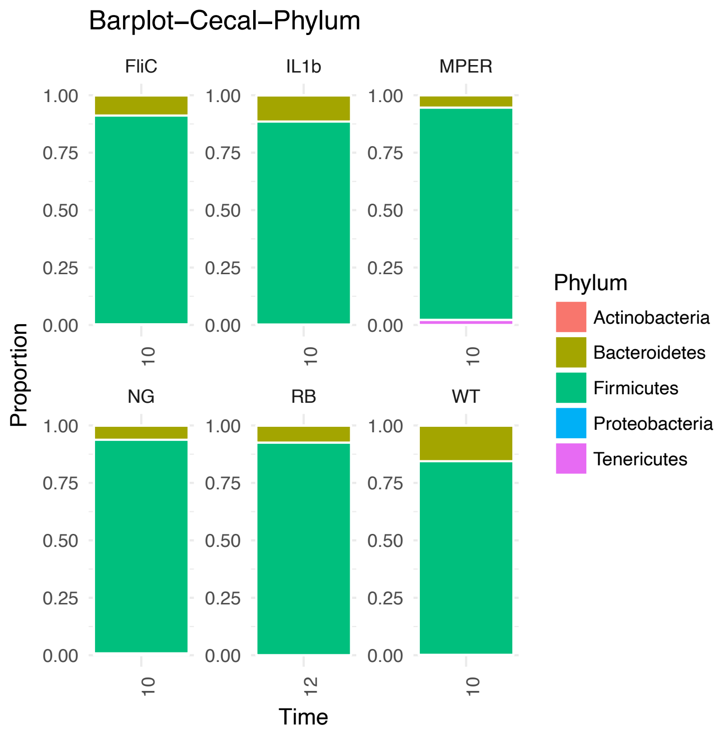

S3 Fig. Bar plots representing the phylum level taxonomic distribution per treatment level per time point of the cecal samples.
