# Supplementary material for: Impact of oral probiotic Lactobacillus acidophilus vaccine strains on the immune response and gut microbiome of mice
Source: PLoS One. 2019 Dec 12;14(12):e0225842. doi: 10.1371/journal.pone.0225842 (PMC6907787; doi:10.1371/journal.pone.0225842)
Supplement: S4 Fig — (PDF) [file pone.0225842.s004.pdf]

A-Fecal

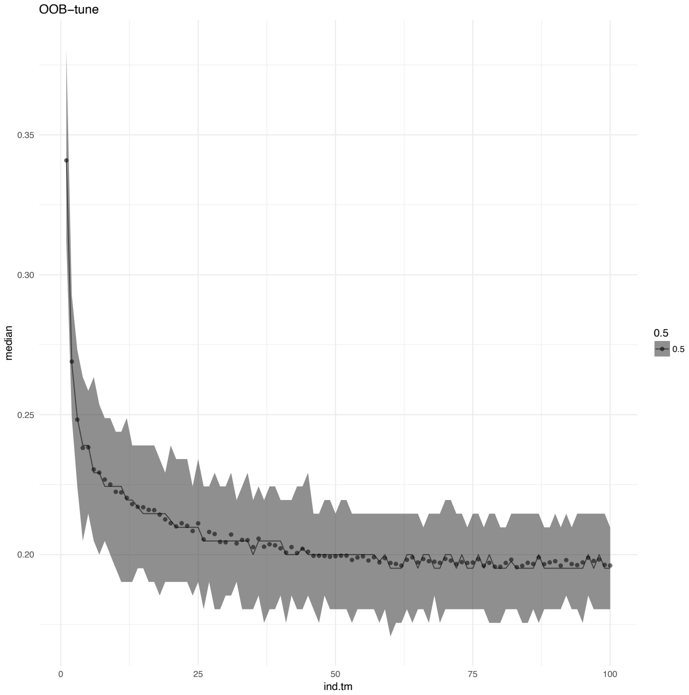

B-Cecal

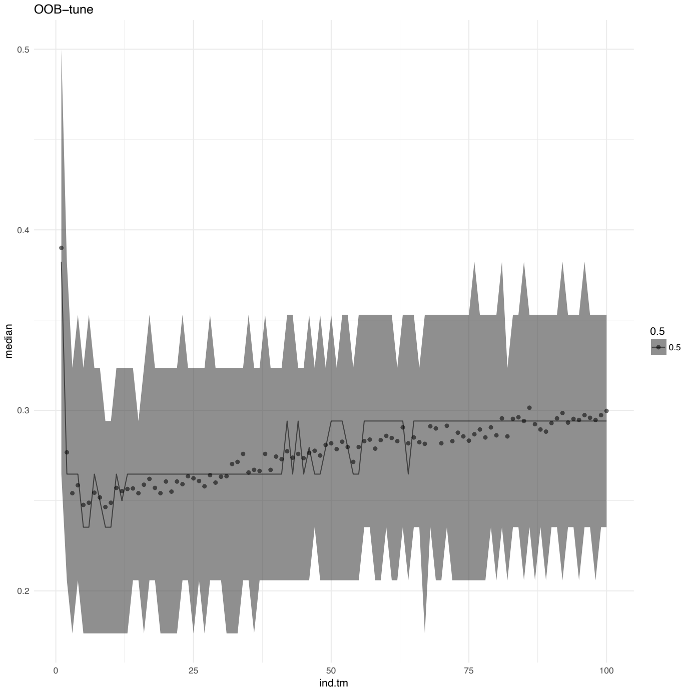

S4 Fig. The out of bag (OOB) tuning plots used to select the optimal number of variables to use in the random forest algorithm. First minimum OOB for fecal samples was observed when using 53 variables and 9 for cecal samples.
