# Supplementary material for: Impact of oral probiotic Lactobacillus acidophilus vaccine strains on the immune response and gut microbiome of mice
Source: PLoS One. 2019 Dec 12;14(12):e0225842. doi: 10.1371/journal.pone.0225842 (PMC6907787; doi:10.1371/journal.pone.0225842)
Supplement: S5 Fig — (PDF) [file pone.0225842.s005.pdf]

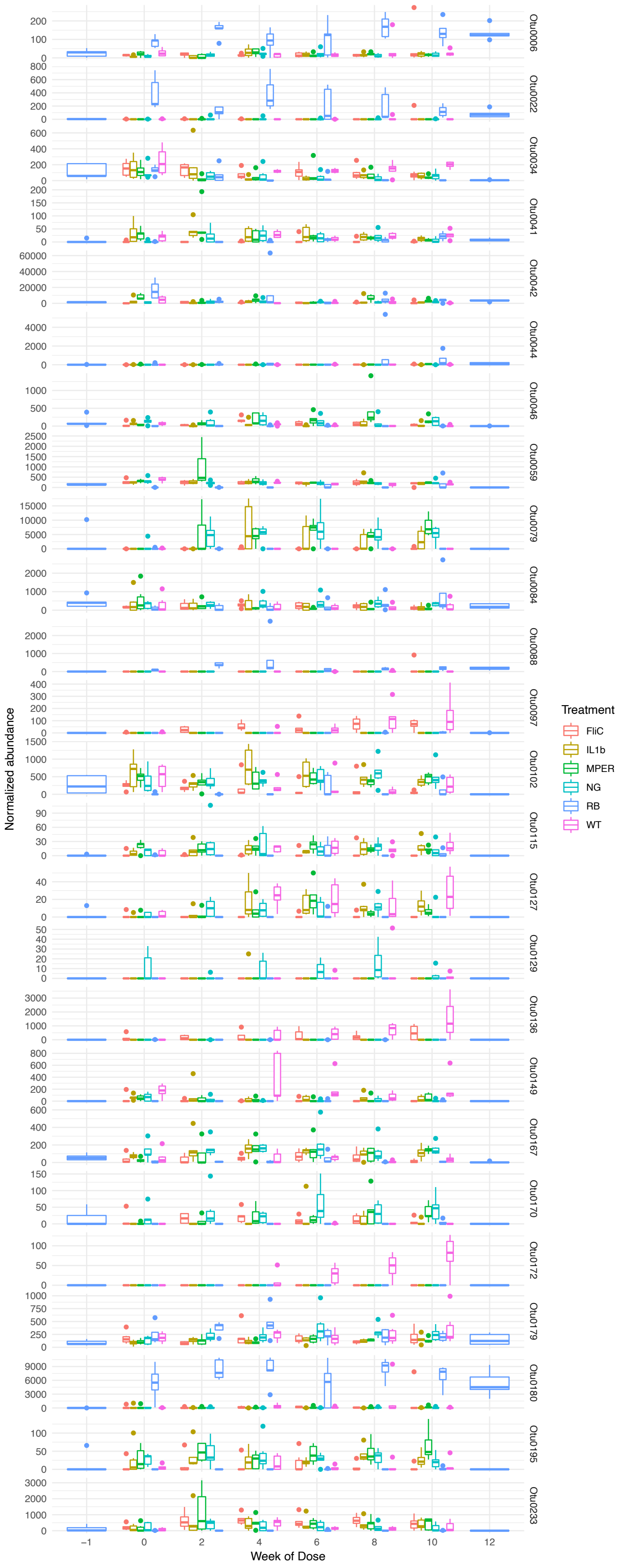

S5 Fig. The normalized abundance of the 25 most impactful OTUs, after the five introduce in Figure 6, associated with the fecal samples.
