# Supplementary material for: Impact of oral probiotic Lactobacillus acidophilus vaccine strains on the immune response and gut microbiome of mice
Source: PLoS One. 2019 Dec 12;14(12):e0225842. doi: 10.1371/journal.pone.0225842 (PMC6907787; doi:10.1371/journal.pone.0225842)
Supplement: S6 Fig — (PDF) [file pone.0225842.s006.pdf]

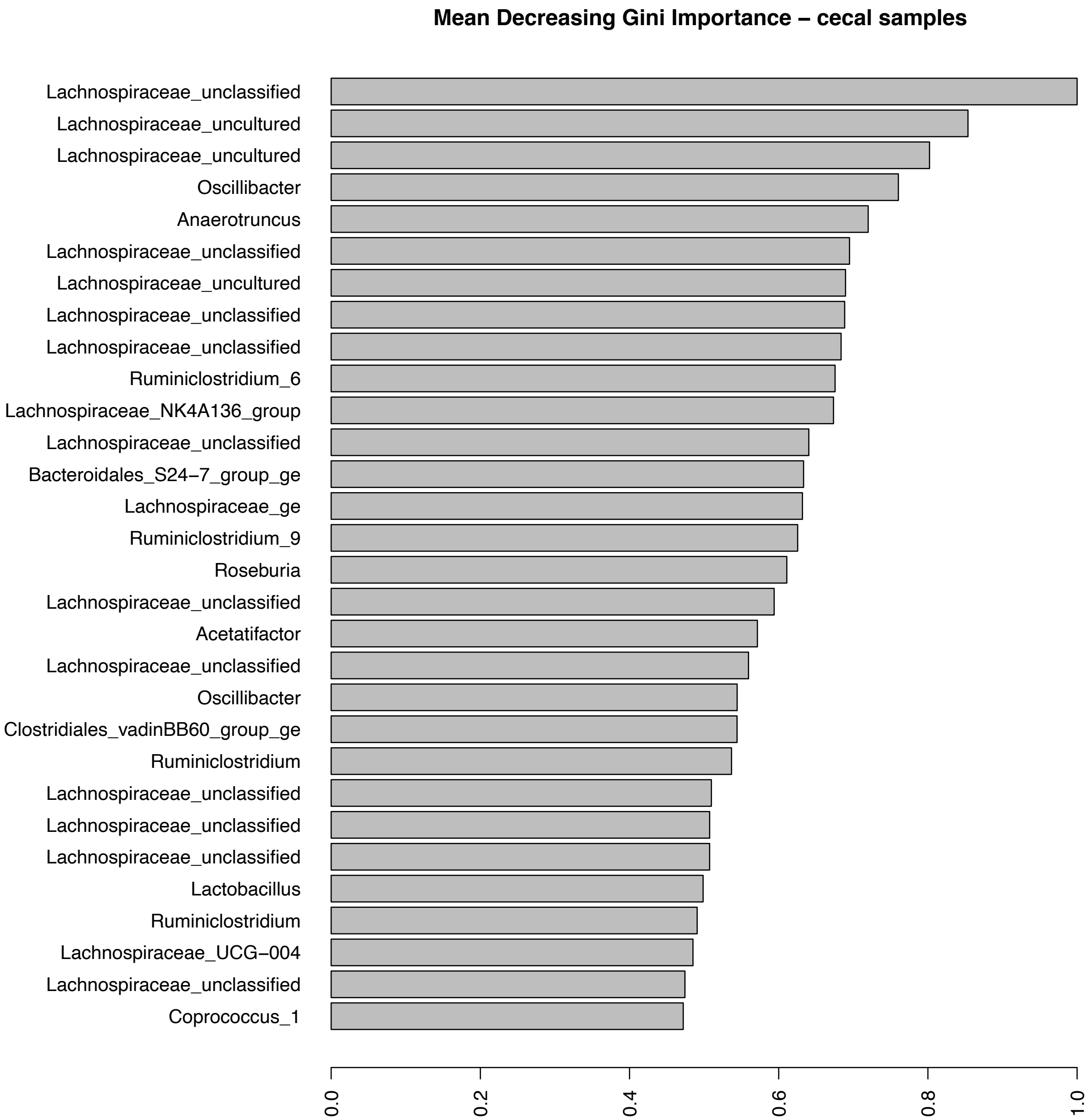

S6 Fig.: The Mean Decreasing Gini OTU importance plot for the cecal samples. X-axis represents the Gini importance measure where high values represent high impact of the OTUs presented on the y-axis.
