# Supplementary material for: Impact of oral probiotic Lactobacillus acidophilus vaccine strains on the immune response and gut microbiome of mice
Source: PLoS One. 2019 Dec 12;14(12):e0225842. doi: 10.1371/journal.pone.0225842 (PMC6907787; doi:10.1371/journal.pone.0225842)
Supplement: S7 Fig — (PDF) [file pone.0225842.s007.pdf]

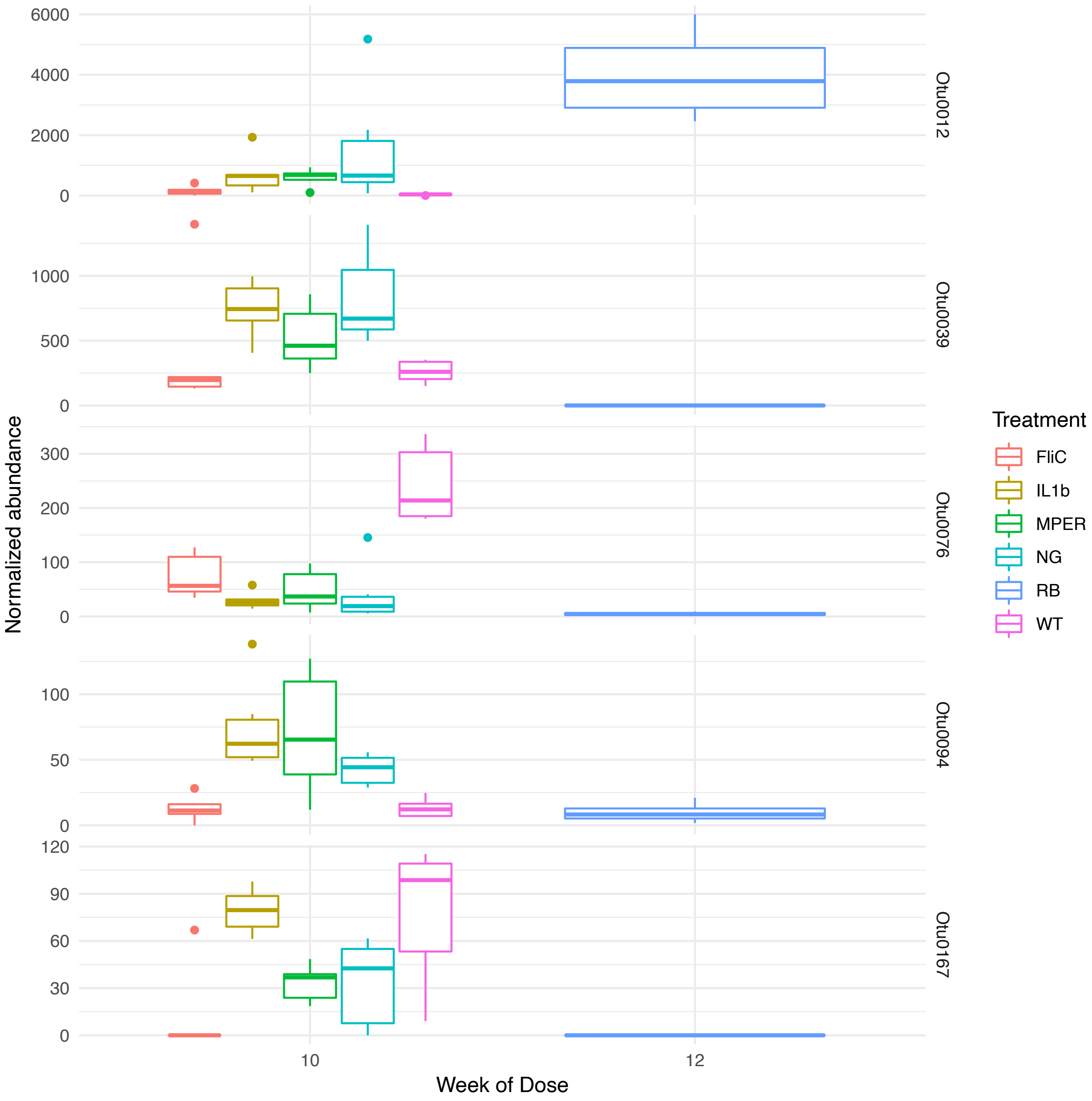

S7 Fig. The normalized abundance of the five most impactful OTUs associated with the cecal samples as observed over time: OTU0076, OTU0094 and OTU0039 were unclassified or uncultured Lachnospiraceae; OTU0012 belonged to the Lachnospiraceae_NK4A136_group genus; and OTU0167 belonged to the Oscillibacter genus.
