# Supplementary material for: Impact of oral probiotic Lactobacillus acidophilus vaccine strains on the immune response and gut microbiome of mice
Source: PLoS One. 2019 Dec 12;14(12):e0225842. doi: 10.1371/journal.pone.0225842 (PMC6907787; doi:10.1371/journal.pone.0225842)
Supplement: S9 Fig — (PDF) [file pone.0225842.s009.pdf]

A

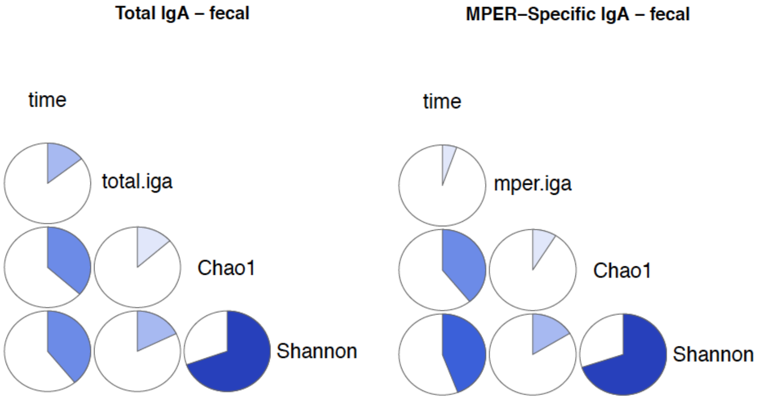

B

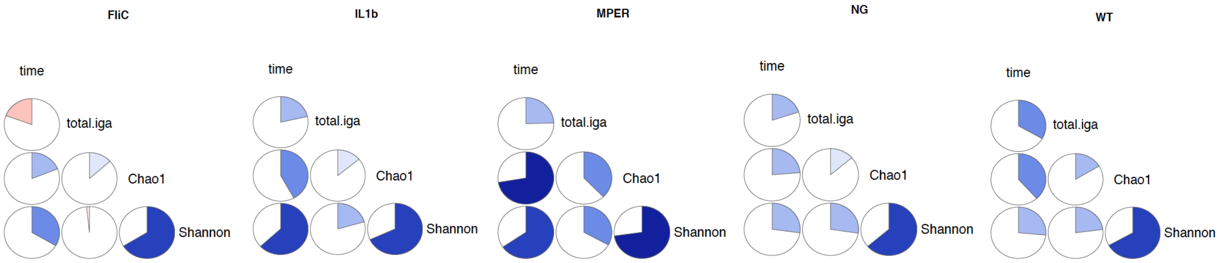

C

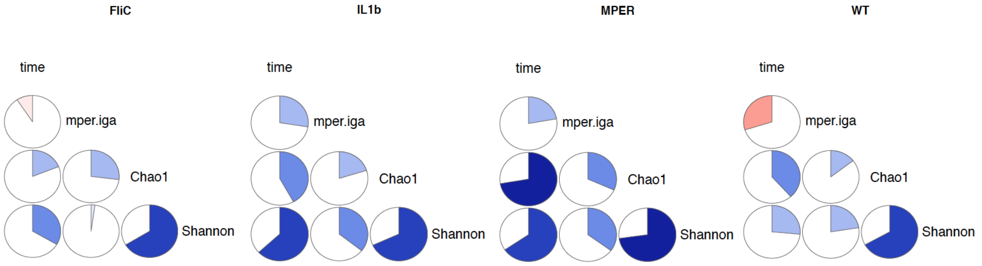

S9 Fig. Pie charts representing Spearman correlation between the Chao1 richness, Shannon diversity, time and total IgA and MPER-specific IgA, separately, for both the (A) combined data and per treatment for (B) total-IgA and (C) MPER-specific samples.
