# Supplementary material for: Impact of oral probiotic Lactobacillus acidophilus vaccine strains on the immune response and gut microbiome of mice
Source: PLoS One. 2019 Dec 12;14(12):e0225842. doi: 10.1371/journal.pone.0225842 (PMC6907787; doi:10.1371/journal.pone.0225842)
Supplement: S10 Fig — (PDF) [file pone.0225842.s010.pdf]

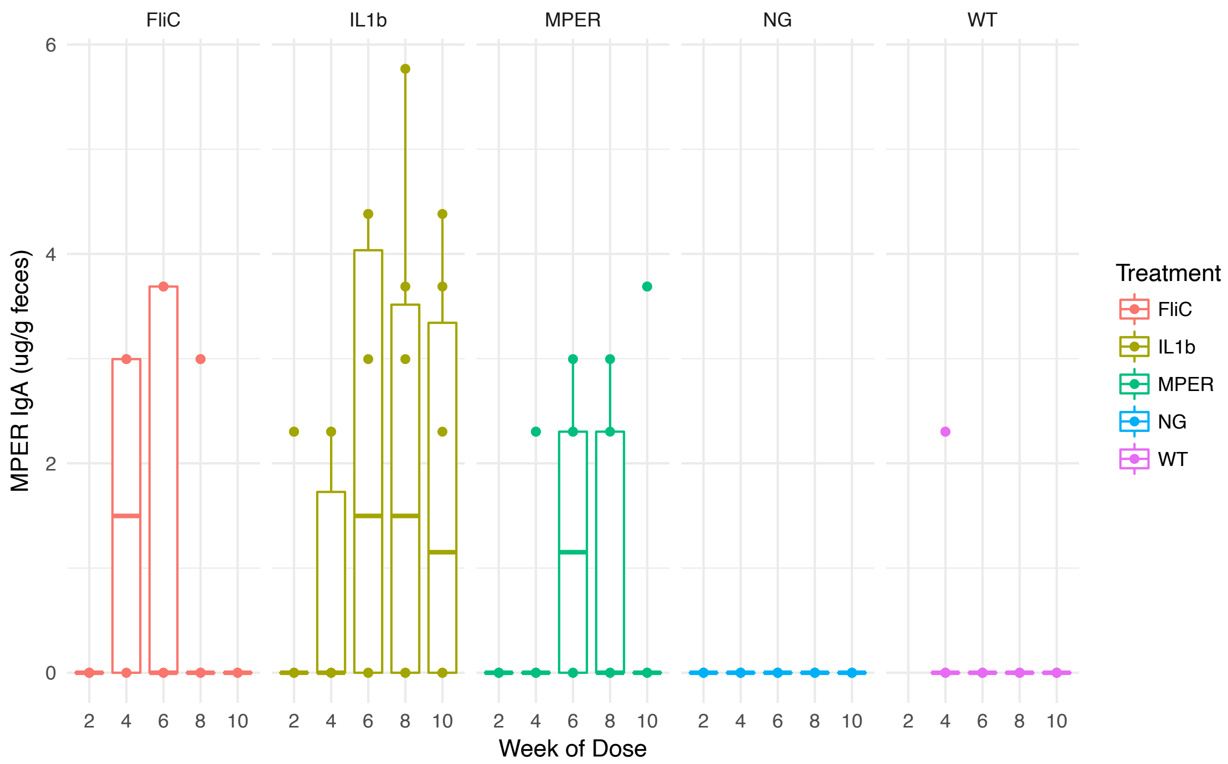

S10 Fig. Box and whisker plots the MPER-specific IgA per time point (0, 2, 4, 6, 8 and 10) under each treatment level. Centerlines represent the median MPER-specific IgA per time point.
