# Supplementary material for: Impact of oral probiotic Lactobacillus acidophilus vaccine strains on the immune response and gut microbiome of mice
Source: PLoS One. 2019 Dec 12;14(12):e0225842. doi: 10.1371/journal.pone.0225842 (PMC6907787; doi:10.1371/journal.pone.0225842)
Supplement: S11 Fig — (PDF) [file pone.0225842.s011.pdf]

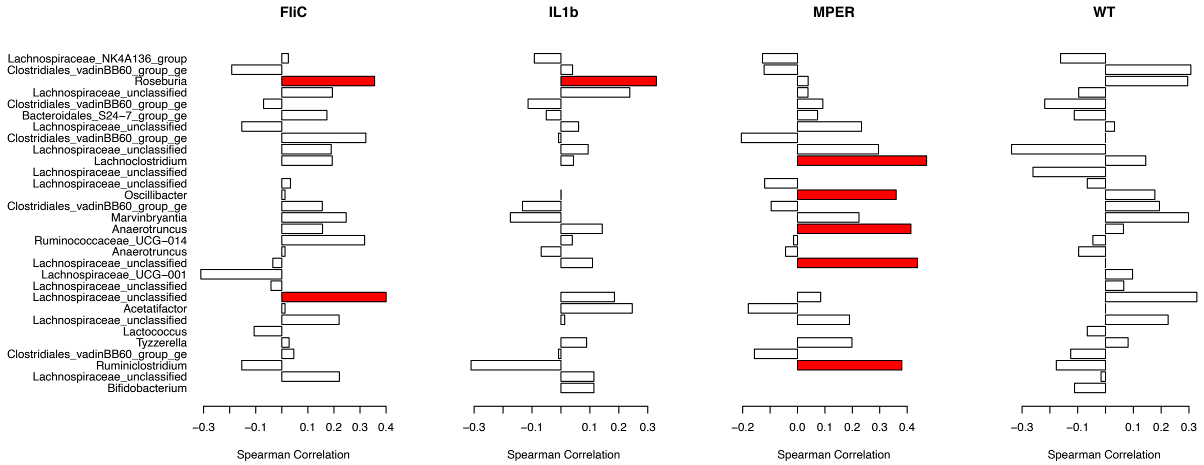

S11 Fig. Spearman correlation plots linking the 30 most impactful taxa observed with the fecal samples and MPER-specific IgA per treatment. Red and blue represent significant positive and negative correlations, respectively, at the 0.1 level of significance with no correction for multiple testing.
