# Supplementary material for: Impact of oral probiotic Lactobacillus acidophilus vaccine strains on the immune response and gut microbiome of mice
Source: PLoS One. 2019 Dec 12;14(12):e0225842. doi: 10.1371/journal.pone.0225842 (PMC6907787; doi:10.1371/journal.pone.0225842)
Supplement: S12 Fig — (PDF) [file pone.0225842.s012.pdf]

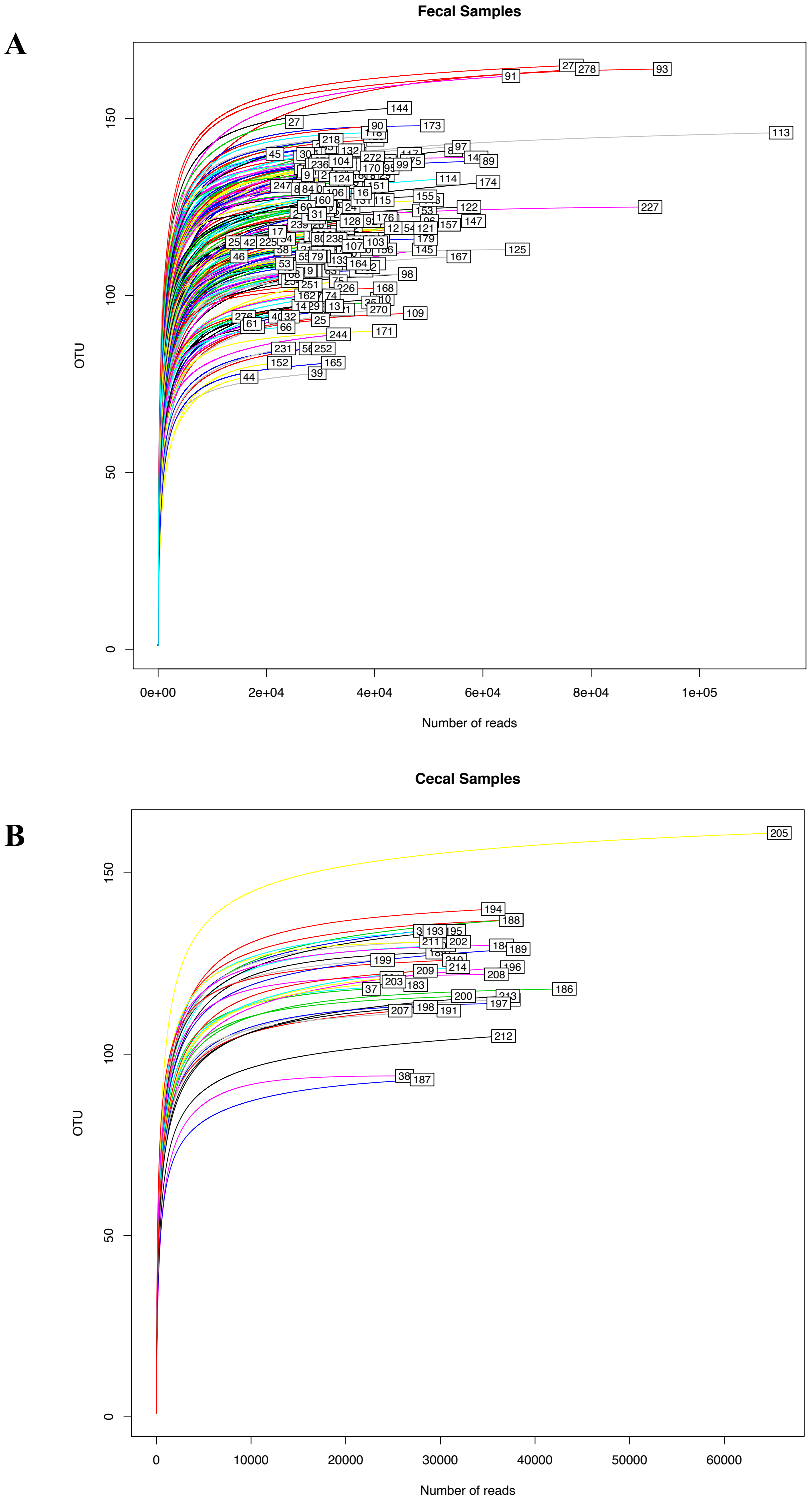

S12 Fig. Rarefaction curves describing depth of sequence coverage for both (A) fecal and (B) cecal samples.
