# Supplementary material for: Impact of oral probiotic Lactobacillus acidophilus vaccine strains on the immune response and gut microbiome of mice
Source: PLoS One. 2019 Dec 12;14(12):e0225842. doi: 10.1371/journal.pone.0225842 (PMC6907787; doi:10.1371/journal.pone.0225842)
Supplement: S13 Fig — (PDF) [file pone.0225842.s013.pdf]

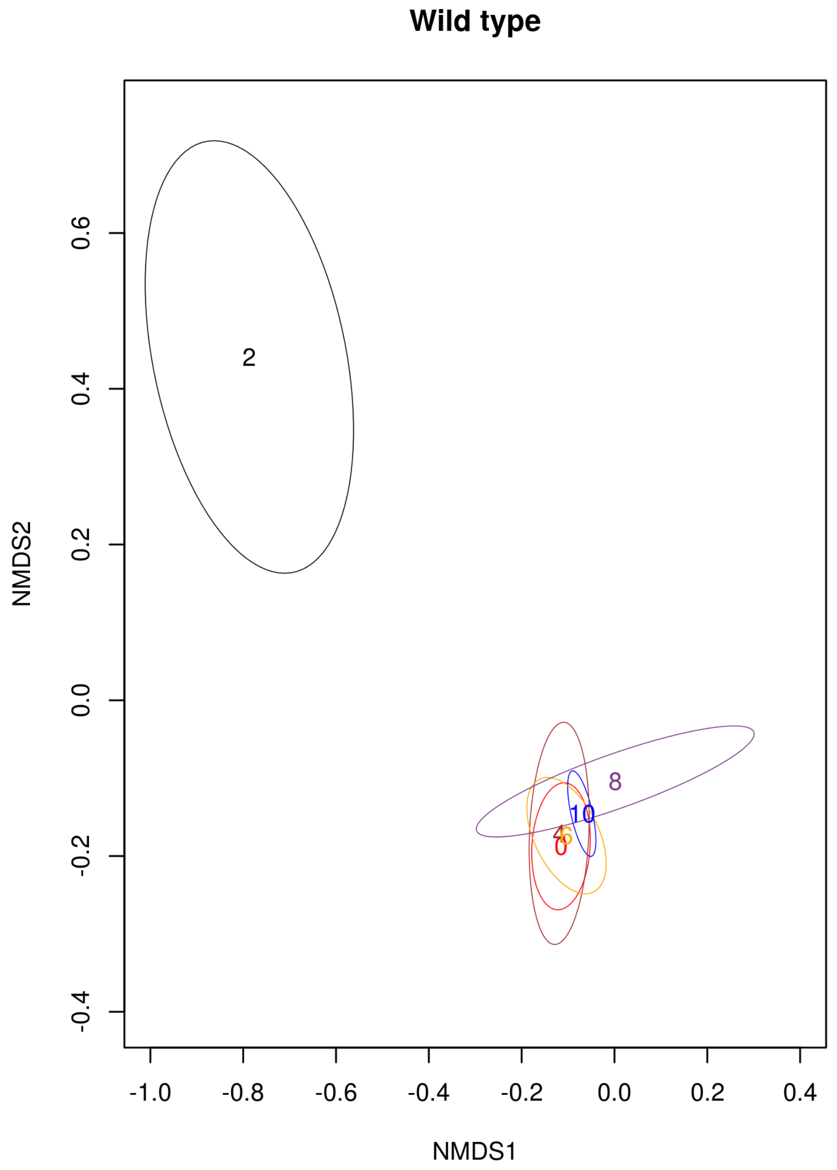

S13 Fig. Nonmetric multidimensional scaling plot identifying the outlier group at time period 2 of the wild type (WT) treatment. The figure clearly shows that the sampled microbiome at time period 2 separates completely from all other time points. Further investigation of these data points indicated that samples were sampled directly after introduction of the probiotic at that time point resulting in over-dominance of the lactobacillus genera. This deviates from our sampling procedure used through the experiment, hence, these samples were dropped from further analyses.
