# Supplementary material for: Impact of oral probiotic Lactobacillus acidophilus vaccine strains on the immune response and gut microbiome of mice
Source: PLoS One. 2019 Dec 12;14(12):e0225842. doi: 10.1371/journal.pone.0225842 (PMC6907787; doi:10.1371/journal.pone.0225842)
Supplement: S1 Table — (DOCX) [file pone.0225842.s014.docx]

**Table S1:** DIC-optimal models for Chao1 richness and Shannon diversity. *Y_k(i)t_* is the observed diversity for mouse *k* nested within treatment *i* at time *t*. *μ* represents the intercept, *β_1_* is the slope for the linear time component *t* and *β_2_* is the slope for the second order time component *t^2^*. *ε* is the error term. Further model details are in the Supplementary File 2.

| Alpha Diversity | DIC Optimal Model |
| --- | --- |
| Chao1 | $Y_{k\left( i \right)t}=\mu_{k\left( i \right)}+\beta_{1k\left( i \right)}t+\beta_{2k\left( i \right)}t^{2}+\varepsilon_{k\left( i \right)t}$ |
| Shannon | $Y_{k\left( i \right)t}=\mu_{k\left( i \right)}+\beta_{1}t+\varepsilon_{i\left( k \right)t}$ |
