# Supplementary material for: Impact of oral probiotic Lactobacillus acidophilus vaccine strains on the immune response and gut microbiome of mice
Source: PLoS One. 2019 Dec 12;14(12):e0225842. doi: 10.1371/journal.pone.0225842 (PMC6907787; doi:10.1371/journal.pone.0225842)
Supplement: S1 Appendix — (DOCX) [file pone.0225842.s022.docx]

**S1 Appendix. Results of data processing and bioinformatics**

**Data Processing and Bioinformatics:**

Using mothur-MiSeq data processing SOP (described in *Materials and Methods*) we identified 1,405 putative OTUs (range 100-249 OTUs per sample) prior to data filtering. Sequencing depth per sample ranged between 8,592 and 115,713 reads. The sequencing error rate was calculated to be 2.414 x 10^-7^ with 89 identified putative OTUs within the mock community samples. Given that the average number of reads associated with the mock community samples was 33,505 and allowing for an error of read-OTU misidentification of 1 in 1000 reads we set a cutoff of 4 (rounding up from 3.3) as the minimum number of reads per OTU required to consider that OTU to be correctly identified. This cutoff resulted in a reduction in the number of OTUs identified in association with the mock community to 15 from 89 (true total is eight). Six of the erroneously identified OTUs belonged in low abundance to only one of the five mock community samples. This also restricted the number of taxa identified as present in the negative control with no sample to four with number of reads less than or equal to 10 in five of these samples and in the negative control with no template to two OTUs with number of reads less than or equal to 4 in three of the four samples. This cutoff was subtracted from all OTU counts per sample resulting in a conservative, total number of putative OTUs of 271 (range 53-165 OTUs per sample) in 245 samples associated with the above described experimental design (including both fecal and cecal samples). S12 Fig shows the resulting rarefaction curves for the fecal and cecal samples, separately, and indicates adequate depth of sequencing to capture sample diversity.

In further assessing the quality of the outcome data we generated NMDS plots separately per treatment level to evaluate time trends and possible outliers. This preliminary data exploration resulted in identification of all samples associated with the positive control (WT) at the second time point as being outliers. These samples grouped together but were quite distant from all other samples belonging to all other time points within that treatment (S13 Fig). In further investigating these samples we concluded that they were sampled directly after introduction of the probiotic at that time point resulting in over-dominance of the lactobacillus genera. This deviates from our sampling procedure used through the experiment (described above); hence, these samples were dropped from further analyses.
