# Supplementary material for: Impact of oral probiotic Lactobacillus acidophilus vaccine strains on the immune response and gut microbiome of mice
Source: PLoS One. 2019 Dec 12;14(12):e0225842. doi: 10.1371/journal.pone.0225842 (PMC6907787; doi:10.1371/journal.pone.0225842)
Supplement: S2 Appendix — (PDF) [file pone.0225842.s023.pdf]

S2 Appendix: Univeriate Models for Analysis of Diversity and Total-IgA Data

Rationale

The experimental design involved in this project includes six treatment levels (WT, MPER, IL1b, FliC, RB and NG) as described in the methods. We consider these treatment levels to be fixed effects that we intend to compare. Nested within these treatment levels we have 6 mice (5 for RB) sampled over 10 time points. Accordingly, we have a repeated measurement/time series design with mice taken as random effects. We evaluate a linear, quadratic and cubic time trend. In what follows we focus on Alpha diversity measures specifically Chao1 richness and Shannon diversity; we use similar models for both and for Total-IgA. For any model that accounts for trend associated with time we include all lower terms in that model when a higher term is included. For example, models that include a cubic term will also include both the quadratic and linear terms. Those models that include a quadratic term will also include the linear one. We use the Deviance Information Criaterlion (DIC) to assess and identify the best models that fit the data as described in the *Methods*. In the case of the cecal samples, where there is no trend over time, we only compare the treatment effects.

Model for trends in alpha diversity

Let y_{itj} be the observed diversity measure for mouse j at time t nested within treatment i. Following is the full model that includes all time trends assuming that each mouse j nested within each treatment i (shown as $i[j]$) follows its own separate cubic time trend:

$$\begin{aligned}
y_{itj} &= \mu_{i[j]} + \beta_{1i[j]}t + \beta_{2i[j]}t^2 + \beta_{3i[j]}t^3 + \varepsilon_{ijt} \\
\mu_{i[j]} &\sim N\left(\mu_i, \sigma^2\right) \\
\beta_{1i[j]} &\sim N\left(\beta_{1i}, \sigma_1^2\right) \\
\beta_{2i[j]} &\sim N\left(\beta_{2i}, \sigma_2^2\right) \\
\beta_{3i[j]} &\sim N\left(\beta_{3i}, \sigma_3^2\right) \\
\varepsilon_{ijt} &\sim N\left(0, \sigma_\varepsilon^2\right) \\
\mu_i &\sim N(0, 1000) \\
\beta_1 i &\sim N(0, 1000) \\
\beta_2 i &\sim N(0, 1000) \\
\beta_3 i &\sim N(0, 1000) \\
\sigma^2 &\sim UNIF(0, 1000) \\
\sigma_1^2 &\sim UNIF(0, 1000) \\
\sigma_2^2 &\sim UNIF(0, 1000) \\
\sigma_3^2 &\sim UNIF(0, 1000) \\
\sigma_\varepsilon^2 &\sim UNIF(0, 1000)
\end{aligned}$$

Where $N(m,n)$ represents a normal distribution with mean m and variance n, $UNIF(a,b)$ represents a uniform distribution with boundaries a and b, $\mu_{i[j]}$ is the intercept associated with each mouse j nested within treatment i, $\beta_{1i[j]}$, $\beta_{2i[j]}$ and $\beta_{3i[j]}$ are the coefficients associated with the linear, quadratic and cubic trends per mouse j nested within treatment i. μ_i, β_{1i}, β_{2i} and β_{3i} are the intrecept, linear coefficient, quadrtatic coefficient and cubic coefficient, repsectively, associated with treatment i and these are the parameters of intrest and have been used to plot the trends in the figures. the σ's repesent the variances.

The model above could be resuced in 29 different ways (resulting in 30 models to compare) that restrict the trend to quadratic or linear and that restrict the model to represent the treatments rather than accounting for the individual random effects of each mouse. For eaxample, a model that accounts for quadratic trend per treatment level is as follows,

$$\begin{aligned}
y_{itj} &= \mu_i + \beta_{1i}t + \beta_{2i}t^2 + \varepsilon_{ijt} \\
\varepsilon_{ijt} &\sim N\left(0, \sigma_\varepsilon^2\right) \\
\mu_i &\sim N(0, 1000) \\
\beta_1 i &\sim N(0, 1000) \\
\beta_2 i &\sim N(0, 1000) \\
\sigma_\varepsilon^2 &\sim UNIF(0, 1000)
\end{aligned}$$

A model that assumes a linear trend shared for all treatments with different intrecept per treatment is as follows,

$$\begin{aligned}
y_{itj} &= \mu_i + \beta_1 t + \varepsilon_{ijt} \\
\varepsilon_{ijt} &\sim N\left(0, \sigma_\varepsilon^2\right) \\
\mu_i &\sim N(0, 1000) \\
\beta_1 &\sim N(0, 1000) \\
\sigma_\varepsilon^2 &\sim UNIF(0, 1000)
\end{aligned}$$

A model assuming no trend associated with time but with mouse specific intercepts is as follows,

$$\begin{aligned}
y_{itj} &= \mu_{i[j]} + \varepsilon_{ijt} \\
\mu_{i[j]} &\sim N\left(\mu_i, \sigma^2\right) \\
\mu_i &\sim N(0, 1000) \\
\sigma^2 &\sim UNIF(0, 1000) \\
\sigma_\varepsilon^2 &\sim UNIF(0, 1000)
\end{aligned}$$

A model that assumes no trend but with intercepts associated with the treatment only (no effect of the mouse) is as follows,

$$
\begin{aligned}
y_{itj} &= \mu_i + \varepsilon_{ijt} \\
\mu_i &\sim N(0, 1000) \\
\sigma_\varepsilon^2 &\sim UNIF(0, 1000)
\end{aligned}
$$

The last two models along with the following simplest null model were used in association with the cecal samples as well as the fecal ones. The minimal null model is as follows,

$$
\begin{aligned}
y_{itj} &= \mu + \varepsilon_{ijt} \\
\mu &\sim N(0, 1000) \\
\sigma_\varepsilon^2 &\sim UNIF(0, 1000)
\end{aligned}
$$

With generalized mean and variance representing no differences between the mice and treatments and no observed time-trend.
